# Diagnostic and prognostic role of circRNAs in pancreatic cancer: a meta-analysis

**DOI:** 10.3389/fonc.2023.1174577

**Published:** 2023-06-08

**Authors:** Ruihua Zhao, Zhuo Han, Haiting Zhou, Yaru Xue, Xiaobing Chen, Xinguang Cao

**Affiliations:** ^1^ Department of Oncology, The First Affiliated Hospital of Zhengzhou University, Zhengzhou, China; ^2^ Department of General Surgery, Tangdu Hospital, The Air Force Medical University, Xi’an, China; ^3^ Department of Oncology, Tongji Hospital, Huazhong University of Science and Technology, Wuhan, China; ^4^ Department of Pediatric Medicine, Northwest Women’s and Children’s Hospital, Xi’an, China; ^5^ Department of Medical Oncology, The Affiliated Cancer Hospital of Zhengzhou University, Zhengzhou, China; ^6^ Department of Digestive Disease, The First Affiliated Hospital of Zhengzhou University, Zhengzhou, China

**Keywords:** pancreatic cancer, diagnostic, prognostic, circular RNAs, meta-analysis

## Abstract

**Background:**

Circular RNAs (circRNAs) are types of endogenous noncoding RNAs produced by selective splicing that are expressed highly specifically in various organisms and tissues and have numerous clinical implications in the regulation of cancer development and progression. Since circRNA is resistant to digestion by ribonucleases and has a long half-life, there is increasing evidence that circRNA can be used as an ideal candidate biomarker for the early diagnosis and prognosis of tumors. In this study, we aimed to reveal the diagnostic and prognostic value of circRNA in human pancreatic cancer (PC).

**Methods:**

A systematic search for publications from inception to 22 July 2022 was conducted on Embase, PubMed, Web of Science (WOS), and the Cochrane Library databases. Available studies that correlated circRNA expression in tissue or serum with the clinicopathological, diagnostic, and prognostic values of PC patients were enrolled. Odds ratios (ORs) and corresponding 95% confidence intervals (CIs) were used to evaluate clinical pathological characteristics. Area under the curve (AUC), sensitivity, and specificity were adopted to assess diagnostic value. Hazard ratios (HRs) were utilized to assess disease-free survival (DFS) and overall survival (OS).

**Results:**

This meta-analysis enrolled 32 eligible studies, including six on diagnosis and 21 on prognosis, which accounted for 2,396 cases from 245 references. For clinical parameters, high expression of carcinogenic circRNA was significantly associated with degree of differentiation (OR = 1.85, 95% CI = 1.47–2.34), TNM stage (OR = 0.46, 95% CI = 0.35–0.62), lymph node metastasis (OR = 0.39, 95% CI = 0.32–0.48), and distant metastasis (OR = 0.26, 95% CI = 0.13–0.51). As for clinical diagnostic utility, circRNA could discriminate patients with pancreatic cancer from controls, with an AUC of 0.86 (95% CI: 0.82–0.88), a relatively high sensitivity of 84%, and a specificity of 80% in tissue. In terms of prognostic significance, carcinogenic circRNA was correlated with poor OS (HR = 2.00, 95% CI: 1.76–2.26) and DFS (HR = 1.96, 95% CI: 1.47–2.62).

**Conclusion:**

In summary, this study demonstrated that circRNA may act as a significant diagnostic and prognostic biomarker for pancreatic cancer.

## 1 Introduction

Pancreatic cancer (PC), a malignant tumor with a poor prognosis, is the seventh leading cause of cancer-related deaths worldwide, with increasing morbidity and mortality (1). Thus, it remains a burden on the international medical landscape. To our knowledge, personal features, environment, lifestyle behaviors, and related primary diseases are the main risk factors (2). Among them, the risk factors related to personal characteristics mostly include sex, age, race, obesity, smoking, heredity, and so on (3, 4). As for environment and lifestyle behaviors, exposure to trace elements (5), smoking (6), excessive alcohol consumption (7), overweight and obesity (8), red meat, and saturated fat increase the probability of pancreatic cancer. In addition, underlying diseases such as diabetes (9), chronic pancreatitis (10), and allergies (11) can also significantly contribute to the risk of pancreatic cancer.

Traumatic surgery is an option for patients with non-metastatic pancreatic cancer. High-end medical equipment and gradually improved surgical techniques significantly reduced patient postoperative mortality (12). In recent years, preoperative adjuvant therapy has also helped patients with pancreatic cancer who had already metastasized before surgery (13). Nonetheless, the overall 5-year survival rate of pancreatic cancer patients was only 2%–9%, which was quite low when compared to other cancers (3). The situation compelled us to reconsider how to increase the rate of pancreatic cancer diagnosis in the early stages to improve patient prognosis.

The use of imaging technologies such as endoscopy or magnetic resonance imaging (MRI) for screening in high-risk populations can significantly improve pancreatic cancer detection rates (14). However, the screening projects have not been widely implemented in China. Because of its high cost and limited sensitivity, imaging technology may not be suitable for early diagnosis, whereas biomarkers have piqued the interest of researchers due to their non-invasive and cost-effective characteristics (15, 16). Carbohydrate antigen199 (CA199) is currently the biomarker used in the clinical diagnosis of pancreatic cancer (17), but an increase in CA199 does not always indicate the presence of cancer, which could be due to biliary infection, inflammation, obstruction, or other diseases (18). The appearance of false positives will reduce the biomarker’s reliability. Although the use of CA199 alone has limitations (19), some studies have found that combining CA199 with other markers such as MUC5AC or microRNA 196a can improve the accuracy of pancreatic cancer diagnosis while still not showing a better result (20, 21).

Recently, new biomarker research on cancer has been emerging endlessly, including microRNA, circRNA, and other novel biomarkers (22, 23). Circular RNAs are a class of long, non-coding RNA molecules that shape a covalently closed continuous loop that has no 5’–3’ polarity and contains no polyA tail (24, 25). The stability of its function is determined by its closed ring structure (26). Previous studies suggested that circRNA may not have biological functions (27), but recent research evidence showed that it may play an important role in the occurrence and development of cancers (28), including liver cancer (29), colorectal cancer (30), breast cancer (31), and so on. The study found that circRNA can adsorb miRNA like a sponge and then affect biological functions; RNA-binding proteins are widely involved in the transcription and translation of proteins (32). They participate in the whole process of circRNA synthesis. CircRNA can also affect the expression of RNA-binding proteins; circRNA can also serve as a template for translation, a cell protein scaffold, and a protein function enhancer (33, 34). Of course, some research teams are also devoted to exploring the function of circRNA in pancreatic cancer. Researchers have detected a variety of circRNAs through microarrays or chips, but only a small part of them confirmed the critical role of their occurrence and development in pancreatic cancer, and the biological process of pancreatic cancer is still under study. The research was still in its initial stages, which did not prevent circRNA from becoming a more valuable biomarker for the diagnosis, treatment, and prognosis of pancreatic cancer in the future (32).

Although the research on circRNA in cancer has been extensively explored, its value in the diagnosis and prognosis of pancreatic cancer has not yet been comprehensively evaluated. The purpose of this study was to evaluate the diagnostic and prognostic value of circRNA in pancreatic cancer and to provide a basis for later functional mechanism research.

## 2 Materials and methods

### 2.1 Search strategy

The study implemented comprehensive searching until 22 July 2022 to identify relevant literature. The resources were primarily from the Pubmed, Embase, Web of Science, Cochrane Library, CNKI, VIP, and Wanfang databases. Meanwhile, the study strictly followed the Preferred Reporting Items for Systematic Review and Meta-analysis (PRISMA) checklist. The search terms were used as follows (1): pancreatic (2); neoplasm OR cancer OR tumor OR neoplastic OR neoplasia OR carcinoma OR malignancy OR malignancies (3); circRNAs* OR circular RNA* (4); diagnosis OR diagnostic OR area under the curve OR AUC OR sensitivity OR specificity OR ROC OR receiver operation characteristic curve OR detection OR screen OR screening; and (5) prognosis OR prognostic OR HR OR Hazard Rate.

### 2.2 Inclusion and exclusion criteria

Enrolled studies were required to meet the following criteria: (1) the subjects of studies were all pancreatic cancer; (2) the study design was case-control or cohort; and (3) the content of studies was associated with circular RNA and diagnosis or prognosis.

The reports were excluded if the studies accorded with one of the following criteria: (1) the topic of studies was not pancreatic cancer or circular RNA; (2) the type of studies was original articles rather than conference papers or books; (3) no valid data could be obtained, such as the sensitivity and specificity of circRNA or HR; and (4) the studies were not written in English.

### 2.3 Data extraction and quality assessment

The data were extracted by two independent individuals. The details were extracted from the included studies as follows: a. first author; b. publish year; c. country; d. ethnicity; e. cancer type; f. circRNA type; g. expression level; h. specimen source; i. no. of patients; j. no. of controls; k. AUC, sensitivity, and specificity (diagnosis); l. follow up time, OS, and HR (prognosis); m. age, gender, tumor size, lymph node metastasis, grade of histology, TNM stage, and distant metastasis (clinical characteristics).

The Quality Assessment for Studies of Diagnostic Accuracy (QUADAS)-2 scale was used to evaluate the quality of the diagnosis studies that were enrolled in this study. Similarly, the Newcastle-Ottawa Scale (NOS) was applied to prognosis studies. The research was considered as high-quality if the scores of the QUADAS scale and NOS all reached higher than six.

### 2.4 Statistical analysis

Stata 15.1 was used to analyze the data. The effect sizes of clinical characteristics (OR) were shown in a forest plot. SROC (summary receiver operator characteristic), pooled sensitivity, and specificity were adopted to evaluate the diagnostic value of circRNA for pancreatic cancer. A pooled hazard ratio (HR) was used to assess the prognosis ability. The random effect model was applied to the merge data. The *I^2^* test and *P*-value were used to check the heterogeneity of the merged results. *I^2^* >50% or *P <*0.05 seemed to indicate strong heterogeneity. Egger’s test was used to examine publication bias quantitatively.

## 3 Results

### 3.1 The process of screening articles

**Figure 1:**
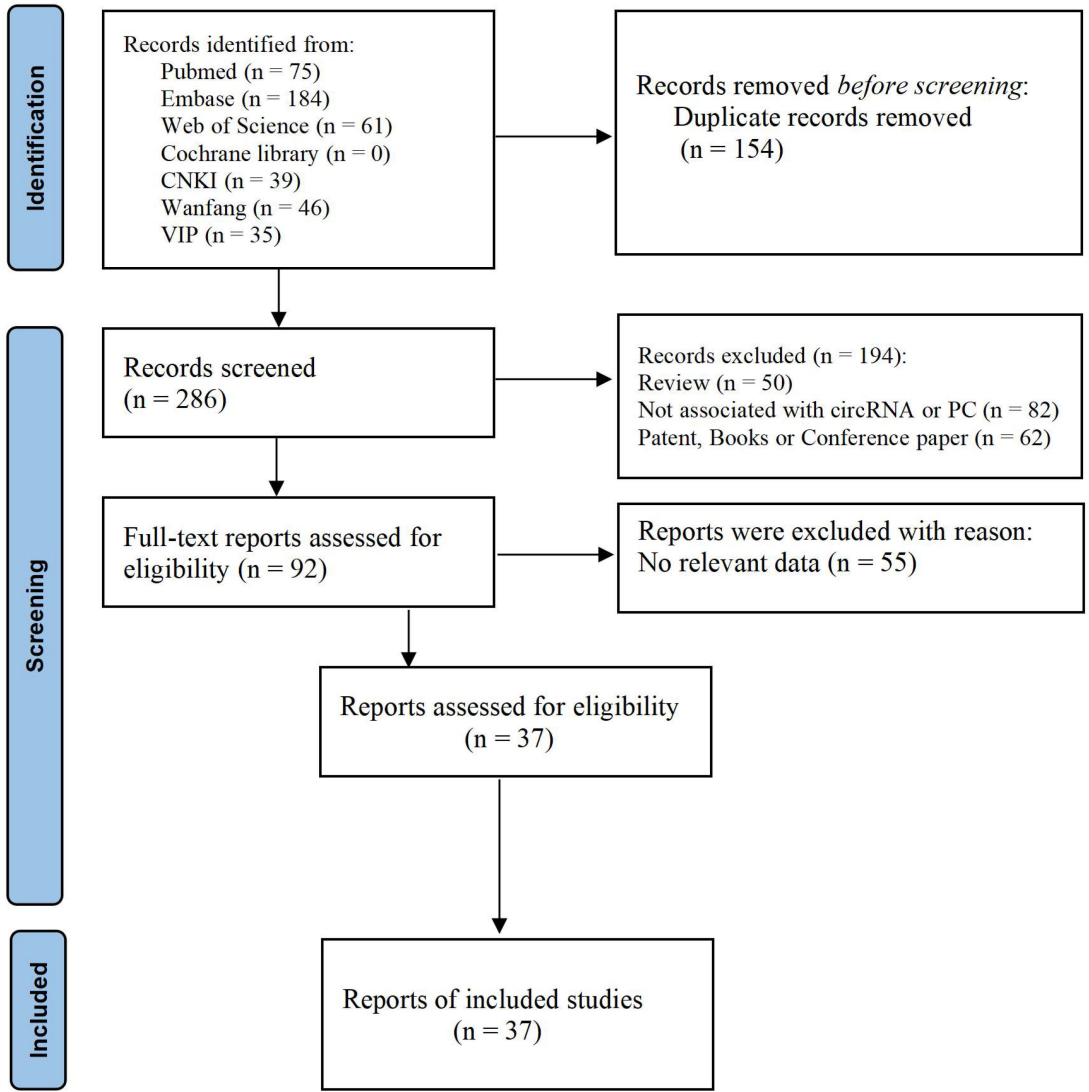
The flow chart shows the process of screening studies.

The detailed process of screening literature is shown in **Figure 1**. The study identified 439 articles through comprehensive searching; however, 154 of them were duplicates. After looking through the titles and abstracts of each article, 195 articles were not considered to be associated with pancreatic cancer or circRNA and were removed. A total of 91 articles were read thoroughly and carefully, and 36 studies were preserved fortunately in the end (35–70). Among them, there are 27 studies related to clinicopathological features, nine studies related to diagnosis, and 24 studies related to prognosis. A total of 2,930 subjects were included, and the publication period of the article was from 2017 to 2022. All studies were carried out in China. Most of the cancer types were pancreatic ductal adenocarcinomas, and the study samples included tissue and serum.

### 3.2 Correlation between clinicopathological characters and circRNA expression level

**Figure 2:**
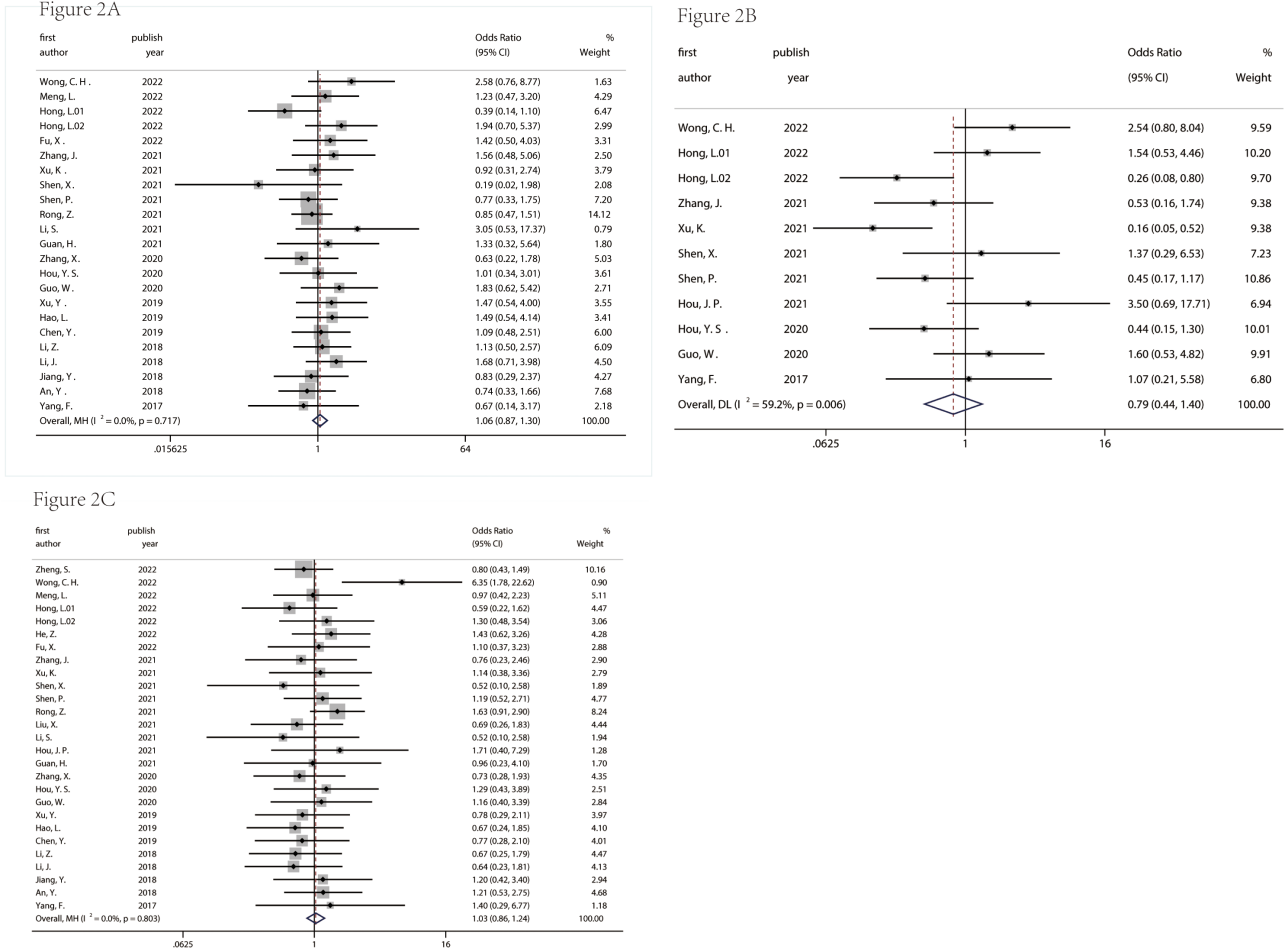
Correlation between age **(A)**, gender **(B)**, tumor size **(C)** and circRNA expression in pancreatic cancer. age: ≥60 years/<60years; gender: male/female; tumor size: ≥5 cm/<5 cm.

**Figure 3:**
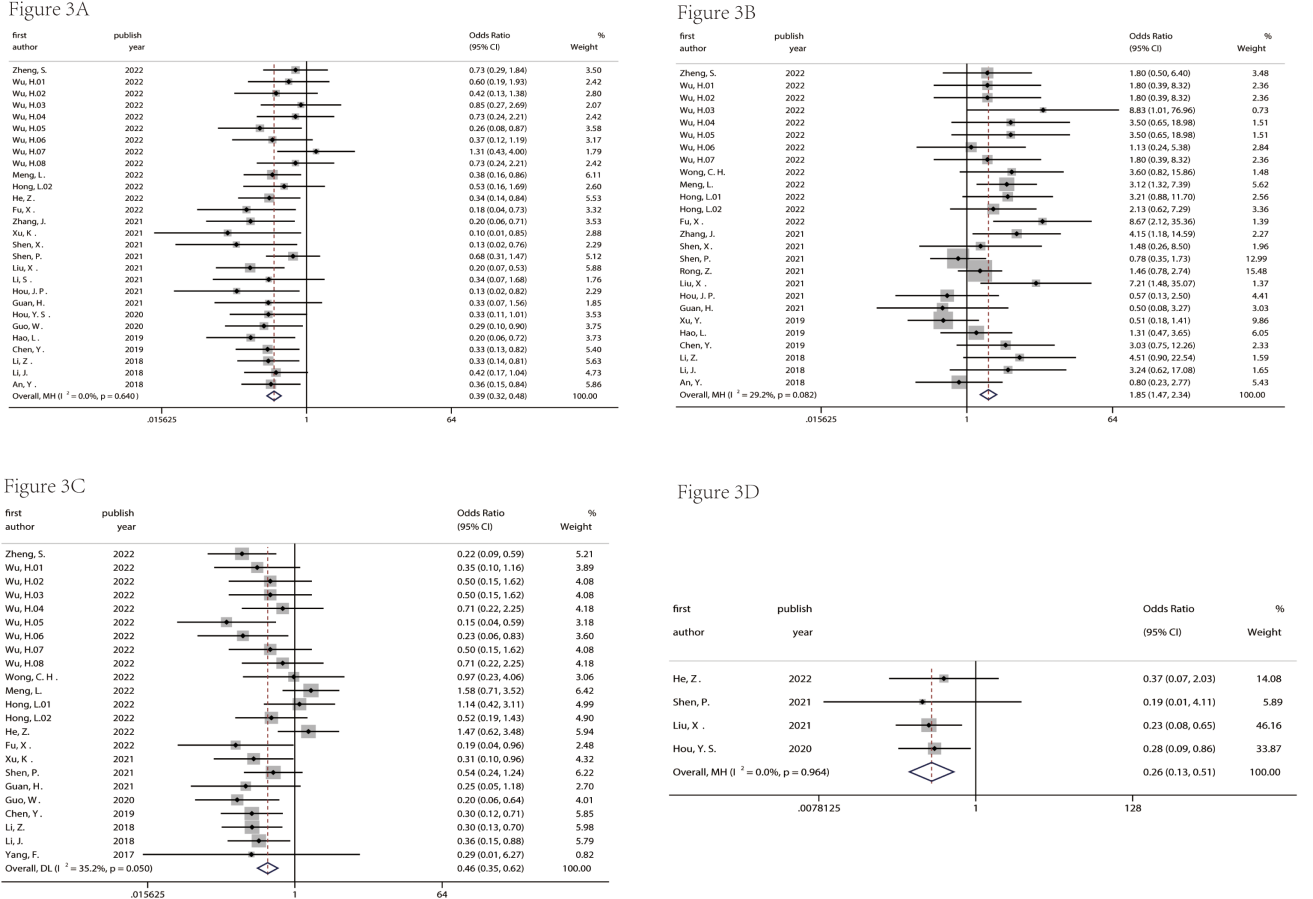
Correlation between lymph node metastasis **(A)**, degree of differentiation **(B)**, TNM stage **(C)**, and distant metastasis **(D)** and circRNA expression in pancreatic cancer.

**Figures 2** and **3** described the correlation between clinicopathological characters and circRNA expression level. The correlation between age, gender, and tumor size with circRNA expression was not found (**Figure 2 and 3**). Interestingly, the results showed that the circRNA expression level was associated with lymph node metastasis (OR = 0.39, 95% CI = 0.32–0.48, **Figure 3A**), degree of differentiation (OR = 1.85, 95% CI = 1.47–2.34, **Figure 3B**), TNM stage (OR = 0.46, 95% CI = 0.35–0.62, **Figure 3C**), and distant metastasis (OR = 0.26, 95% CI = 0.13–0.51, **Figure 3D**). This means that the lower the degree of tumor differentiation, the higher the expression level of circRNA; the occurrence of lymph node metastasis and distant metastasis is also related to the higher expression of circRNA; and the later the TNM stage, the higher the expression level of circRNA. The detailed information on the included studies with clinicopathological characters is in **Supplementary Tables 1–7**.

### 3.3 Diagnosis of circRNA for pancreatic cancer

**Figure 4:**
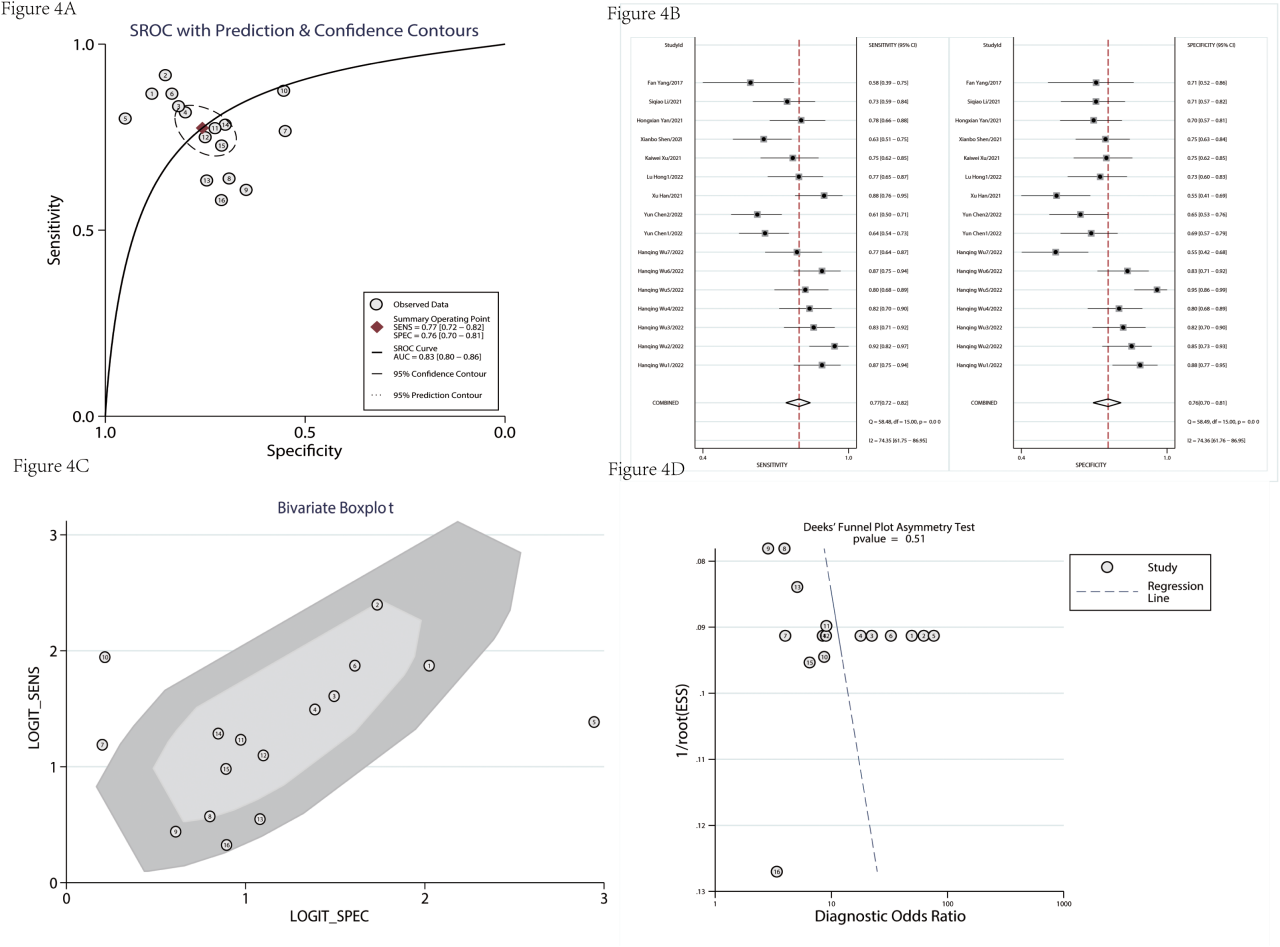
The diagnostic value was evaluated. SROC of enrolled diagnostic studies **(A)**. The pooled sensitivity and specificity of enrolled diagnostic studies **(B)**. The bivariate boxplot of diagnostic studies **(C)**. The funnel plot was used to assess the publication bias of enrolled diagnostic studies **(D)**. SROC, summary receiver operator characteristic.

**Figure 5:**
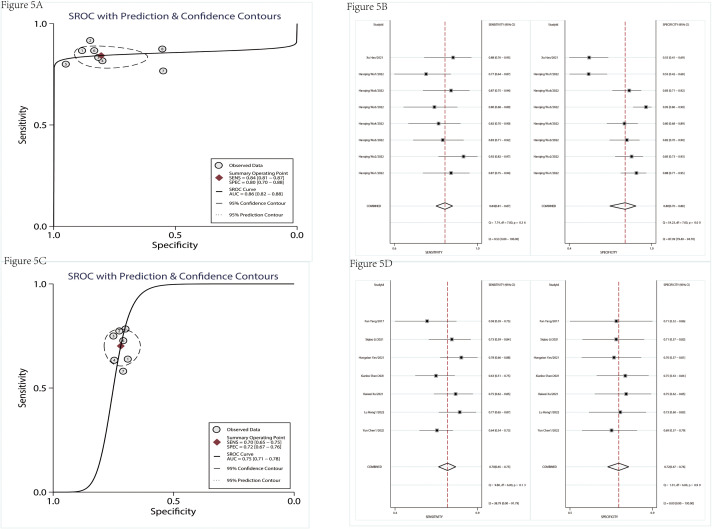
The subgroup analysis of diagnostic studies. SROC of diagnostic studies in tissue **(A)**. The pooled sensitivity and specificity of diagnostic studies in tissue **(B)**. SROC of diagnostic studies in serum **(C)**. The pooled sensitivity and specificity of diagnostic studies in serum **(D)**. SROC, summary receiver operator characteristic.

There were nine studies used to assess the diagnostic value (**Table 1**). **Table 2** indicated that the included studies of diagnosis were high quality. The merged diagnostic performance of circRNA for pancreatic cancer was 0.83 (95% CI = 0.80–0.86) from the SROC curve in **Figure 4A**. However, we also found that the pooled sensitivity and specificity were highly heterogeneous (**Figure 4B**). The bivariate boxplot was used to seek out the sources of heterogeneity. As shown in the bivariate boxplot (**Figure 4C**), two studies were not involved in the boxplot, including studies 5 and 10. The testing sample source in study 5 was tissue, but in study 10, it was plasma, which may indicate that the specimen source was the main factor in heterogeneity. The results of the subgroup analysis showed that if the sample was from tissue, the diagnostic value reached 0.86 (95% CI = 0.82–0.88), and pooled sensitivity and specificity were 84% and 80%, respectively (**Figures 5A, B**). But the diagnostic value of the serum sample (AUC = 0.75, 95% CI = 0.72–0.78) was lower than that of the tissue sample; and the pooled sensitivity and specificity were 70% and 72%, respectively (**Figures 5C, D**). The results of **Figure 4D** indicated that there was no significant publication bias in the literature included in the diagnostic studies (*P* = 0.51).

**Table 1:** Diagnosis studies of circRNA and pancreatic cancer.

first author	publish year	countary	ethnicity	cancer type	circRNA type	expression	specimen source	No.of patients	No.of controls	cutoff value	AUC	TP	FP	FN	TN	sensitivity	specificity	detection method	QUADAS0-2
Hanqing Wu1	2022	China	Asian	PDAC	Hsa_circ_0000515	U	tissue	60	60	1.780	0.920	52	7	8	53	86.70%	88.30%	qRT-PCR	7
Hanqing Wu2	2022	China	Asian	PDAC	Hsa_circ_0000517	U	tissue	60	60	1.422	0.922	55	9	5	51	91.70%	85.00%	qRT-PCR	7
Hanqing Wu3	2022	China	Asian	PDAC	Has circ_0000520	U	tissue	60	60	1.444	0.887	50	11	10	49	83.30%	81.70%	qRT-PCR	7
Hanqing Wu4	2022	China	Asian	PDAC	Hsa_circ_0000514	U	tissue	60	60	1.771	0.899	49	12	11	48	81.70%	80.00%	qRT-PCR	7
Hanqing Wu5	2022	China	Asian	PDAC	Hsa_circ_0011385	U	tissue	60	60	2.587	0.953	48	3	12	57	80.00%	95.00%	qRT-PCR	7
Hanqing Wu6	2022	China	Asian	PDAC	Hsa_circ_0072088	U	tissue	60	60	1.669	0.902	52	10	8	50	86.70%	83.30%	qRT-PCR	7
Hanqing Wu7	2022	China	Asian	PDAC	Hsa_circ_0055033	U	tissue	60	60	1.025	0.697	46	27	14	33	76.70%	55.00%	qRT-PCR	7
Yun Chen1	2022	China	Asian	PC	Has_circ_0141633	U	plasma	97	71	NA	0.700	62	22	35	49	64.32%	68.54%	qRT-PCR	
Yun Chen2	2022	China	Asian	PC	Has_circ_0008234	U	plasma	97	71	NA	0.670	59	25	38	46	60.79%	65.46%	qRT-PCR	
Xu Han	2021	China	Asian	PDAC	Hsa_circ_0071036	U	tissue	56	56	NA	0.650	49	25	7	31	87.50%	55.40%	qRT-PCR	7
Lu Hong1	2022	China	Asian	PDAC	Hsa_circ_0006220	U	plasma	62	62	NA	0.782	48	17	14	45	77.42%	72.58%	qRT-PCR	7
Kaiwei Xu	2021	China	Asian	PC	Hsa_circ_0013587	U	plasma	60	60	NA	0.802	45	15	15	45	75.81%	75.81%	qRT-PCR	7
Xianbo Shen	2021	China	Asian	PC	Hsa_circ_001569	U	plasma	71	71	NA	0.716	45	18	26	53	62.76%	74.29%	qRT-PCR	7
Hongxian Yan	2021	China	Asian	PC	chr14∶101402109-101464448C	U	plasma	60	60	3.230	0.939	47	18	13	42	78.30%	70.00%	qRT-PCR	
Siqiao Li	2021	China	Asian	PC	circ-MFN2	U	plasma	55	55	4.110	0.861	40	16	15	39	72.70%	70.90%	qRT-PCR	
Fan Yang	2017	China	Asian	PDAC	circ-LDLRAD3	U	plasma	31	31	9.315	0.670	18	9	13	22	57.38%	70.49%	qRT-PCR	7

**Table 2:** QUADAS-2 scale of diagnosis studies.

study	Risk of bias				Applicability		
	Patient selection	Index tests	Reference standard	Appropriate interval	Patient selection	Index tests	Reference standard
Hanqing Wu	☺	☺	☺	**?**	☺	☺	☺
Yun Chen	?	?	☺	**?**	☺	☺	☺
Xu Han	?	?	☺	☹	☺	☺	☺
Lu Hong	?	☺	☺	☹	☺	☺	☺
Kaiwei Xu	☺	☺	☺	☹	☺	☺	☺
Xianbo Shen	☺	☺	☺	☹	☺	☺	☺
Hongxian Yan	☺	☺	☺	?	☺	☺	☺
Siqiao Li	?	?	☺	?	☺	☺	☺
Fan Yang	☺	☺	☺	☹	☺	☺	☺

### 3.4 Prognosis of circRNA for pancreatic cancer

**Figure 6:**
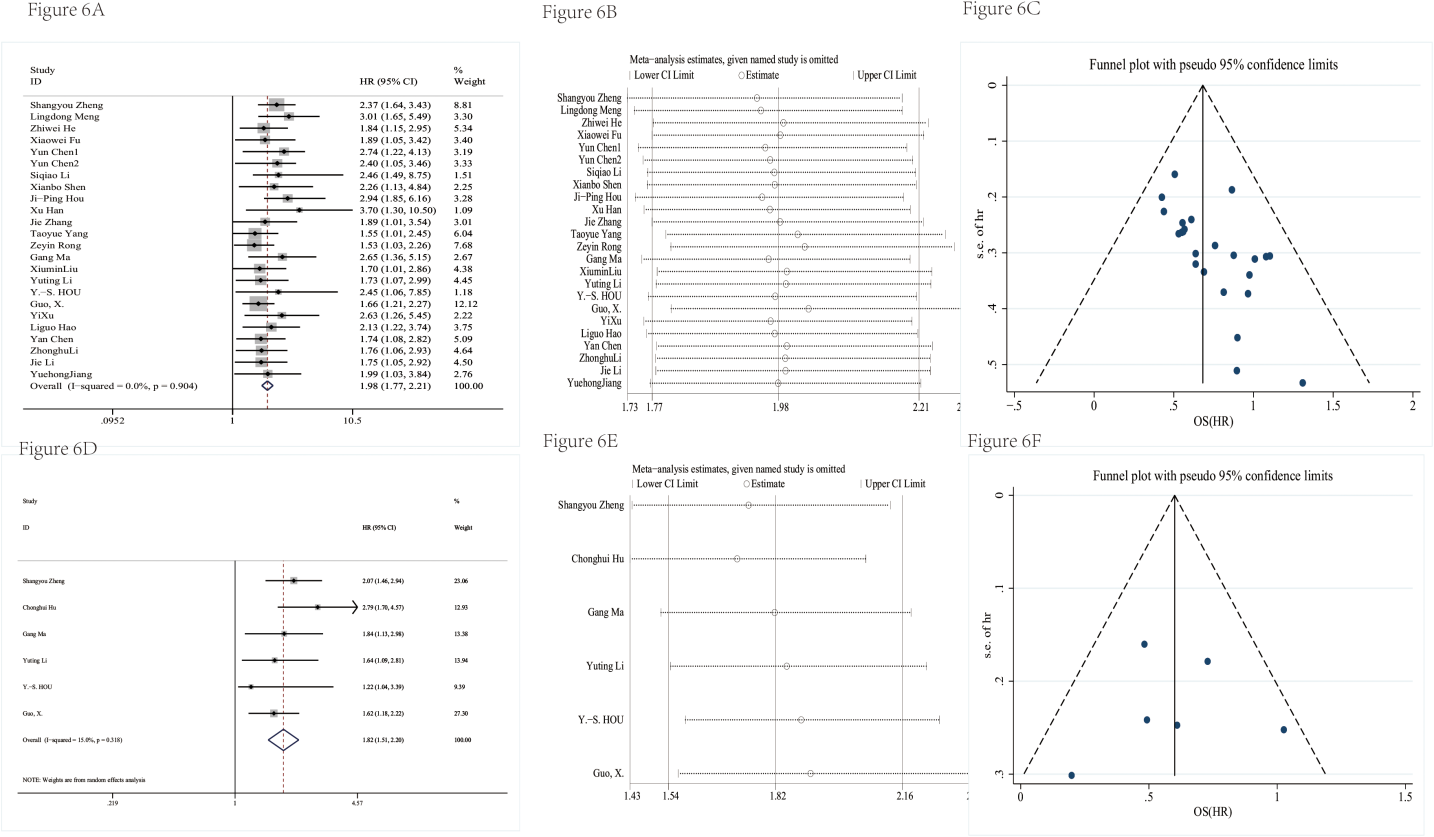
The prognosis value was evaluated. The forest plot had a pooled HR of OS **(A)**. The sensitivity analysis of OS **(B)**. The funnel plot of OS **(C)**. The forest plot had a pooled HR of DFS **(D)**. The sensitivity analysis of DFS **(E)**. The funnel plot of DFS **(F)**.

**Table 3** showed the detailed information of prognosis. There was no significant heterogeneity in the combined hazard ratio (HR). The results of **Figures 6A, D** showed that the high expression level of circRNAs indicated a poor prognosis; the pooled HRs of OS and DFS were 1.98 (95% CI = 1.77–2.21) and 1.82 (95% CI = 1.51–2.20), respectively. The sensitivity analysis showed that no single study could influence results (**Figures 6B, E**), which suggested that circRNA was a more stable biomarker and has some prognostic implications for patients’ DFS or OS. A funnel plot (**Figures 6C, F**) and Egger’s test were used to evaluate publication bias. The *P-*values of Egger’s test were 0.08 for OS and 0.84 for DFS.

**Table 3:** Prognosis studies of circRNA and pancreatic cancer.

first author	publish yearar	country	ethnicity	cancer type	circRNA type	expression	specimen source	high expression	low expression	follow-up time (month)	OS(HR)	LL	UL	DFS(HR)	LL	UL	NOS	
Shangyou Zheng	2022	China	Asian	PDAC	Circ-CUL2	U	tissue	80	81	80	2.374	1.645	3.426	2.073	1.460	2.942	6	high/low
Lingdong Meng	2022	China	Asian	PDAC	Circ-STX6	U	tissue	48	49	80	3.013	1.655	5.486				7	high/low
Zhiwei He	2022	China	Asian	PC	Circ-ATG7	U	tissue	46	46	60	1.840	1.150	2.950				6	high/low
Xiaowei Fu	2022	China	Asian	PC	Hsa_circ_0046523	U	tissue	29	28	60	1.890	1.050	3.420				7	high/low
Chonghui Hu	2022	China	Asian	PDAC	Circ-FARP1	U	tissue	41	41	20				2.790	1.700	4.570	7	high/low
Yun Chen1	2022	China	Asian	PC	Has_circ_0141633	U	plasma	48	49	60	2.740	1.220	4.130				7	high/low
Yun Chen2	2022	China	Asian	PC	Has_circ_0008234	U	plasma	48	49	60	2.400	1.050	3.460				7	high/low
Siqiao Li	2021	China	Asian	PC	Circ-MFN2	U	plasma	48	7	60	2.457	1.489	8.749				6	high/low
Xianbo Shen	2021	China	Asian	PC	Hsa_circ_001569	U	plasma	13	13	60	2.256	1.132	4.836				6	high/low
Ji-Ping Hou	2021	China	Asian	PC	Circ-CCT3	U	tissue	15	15	60	2.940	1.850	6.160				6	high/low
Xu Han	2021	China	Asian	PDAC	Hsa_circ_0071036	U	tissue	90	90	24	3.700	1.300	10.500				7	high/low
Jie Zhang	2021	China	Asian	PC	Hsa_circ_0066147	U	tissue	24	21	60	1.890	1.010	3.540				8	high/low
Taoyue Yang	2021	China	Asian	PDAC	Circ-RHOBTB3	U	tissue	55	55	80	1.550	1.010	2.450				7	high/low
Zeyin Rong	2021	China	Asian	PDAC	Circ-EYA3	U	tissue	138	71	70	1.530	1.030	2.260				6	high/low
Gang Ma	2021	China	Asian	PDAC	Hsa_circ-0005105	U	tissue	38	37	60	2.650	1.360	5.150	1.840	1.130	2.980	7	high/low
XiuminLiu	2021	China	Asian	PDAC	Hsa_circ_00684	U	tissue	40	40	40	1.700	1.010	2.860				7	high/low
Yuting Li	2020	China	Asian	PDAC	Circ-PCAC1	U	Tissue	38	38	60	1.733	1.066	2.991	1.636	1.090	2.811	6	high/low
Y.-S. HOU	2020	China	Asian	PC	Hsa_circ_0005273	U	tissue	23	33	80	2.450	1.060	7.850	1.220	1.040	3.390	8	high/low
Guo, X.	2020	China	Asina	PDAC	Circ-BFAR	U	tissue	104	104	60	1.658	1.213	2.267	1.620	1.183	2.217	8	high/low
YiXu	2019	China	Asian	PDAC	Hsa_circ_0030235	U	tissue	32	30	60	2.626	1.264	5.455				7	high/low
Liguo Hao	2019	China	Asian	PDAC	Hsa_circ_0007534	U	tissue	30	30	60	2.135	1.217	3.745				6	high/low
Yan Chen	2019	China	Asian	PDAC	Circ-ASH2L	U	tissue	45	45	50	1.741	1.075	2.821				6	high/low
Zhonghu Li	2019	China	Asian	PDAC	Circ-PDE8	U	tissue	46	47	50	1.764	1.064	2.925				7	high/low
Jie Li	2018	China	Asian	PDAC	Circ-IRAS	U	tissue	42	43	50	1.749	1.047	2.924				7	high/low
YuehongJiang	2018	China	Asian	PDAC	Hsa_circ_0001649	D	tissue	25	33	60	1.993	1.035	3.837				7	high/low

## 4 Discussion

In this study, a meta-pooling analysis of the 36 included studies showed that circRNA may be a biomarker with great potential for both diagnosis and prognosis of pancreatic cancer, and it was also found that the expression level of circRNA in humans correlated with the degree of tumor differentiation, TNM stage, whether the lymph nodes metastasized, and whether distant metastasis occurred. Using tissues as samples to detect circRNA expression levels may have higher diagnostic power, while using circRNA assays as observational monitoring indicators during the later stages of patient treatment may more positively suggest patient outcomes and long-term prognosis.

The correlation between circRNA expression level in pancreatic cancer and clinicopathological characteristics did not reach a consistent conclusion, so it is necessary to carry out a corresponding analysis. The study found that has_ circ_ 0075829 is highly expressed in pancreatic cancer and is related to tumor size and lymph node metastasis (71). However, the pooled analysis results did not find that tumor size was related to circRNA expression levels. While many studies have shown that the expression of circRNA is related to the occurrence of lymph node metastasis and TNM stage, which is consistent with the conclusion of our study. The high expression of circ BFAR (53) and circ-ASH2L (56) in pancreatic cancer was positively correlated with lymph node metastasis and advanced TNM stage, hsa_ circ_ 0001649 (59) and circ-IARS (58) were related to the degree of tumor differentiation. In the published research, Yang and others found that circ-LDLRAD3 can promote the proliferation, invasion, and migration of tumor cells by acting on the miR-137-3p/PTN axis through functional mechanism research in 2017 (60); in 2018, Li and team also found that circ-PDE8A stimulated the growth of tumor cells by targeting the miR-338/MACC1/MET axis while affecting the liver metastasis of tumor cells (57). The in-depth study of the mechanism also suggested that the high expression of circRNA in tumor tissue would lead to a poor prognosis, which was confirmed by the results of the survival analysis of patients. Zheng found in the 2022 study that the higher the expression level of circCUL2 in tumor tissue, the shorter the disease-free survival and total survival time of PDAC patients (35).

Although the current research results believe that circRNA is a diagnostic tool for pancreatic cancer, there is a greater correlation between the size of the diagnostic efficacy and the samples tested. This study found that the diagnostic ability of tissue samples is significantly higher than that of serum samples, which is consistent with the result that the expression level of circRNA in tissues is higher than that of serum. However, compared with the stability of the biomarkers in the serum, the preoperative tissue samples submitted for examination in clinical practice have greater heterogeneity, which has a great relationship with the technical level of the sending doctors and the tumor location. Previous studies have shown that circRNA is abundant in serum exosomes (72). Therefore, the research on tumor biomarkers in serum is still worth exploring, and the focus is on serum tumor markers with strong diagnostic ability.

This study is the first to comprehensively analyze the correlation between circRNA expression in pancreatic cancer and clinicopathological characteristics, as well as its potential diagnostic and prognostic tool for pancreatic cancer in Chinese population, which is one of the greatest advantages of this study. Secondly, this study conducted a comprehensive analysis of the functions of circRNA in the diagnosis, treatment, and prognosis of pancreatic cancer patients, which may prove that circRNA, as an emerging biomarker, may have greater potential in the future. However, there are also some deficiencies in this study. For example, for diagnostic research, tissue as a sample shows greater diagnostic potential than serum. Although we have tried our best to comprehensively screen high-quality research, the amount of literature that can be included in both is relatively small, which may indicate that the direction of our future research needs to be improved. There was some publication bias in the pooled OS in prognostic studies, which may be because published studies prefer positive results, but there was no doubt about circRNA as a prognostic biomarker for pancreatic cancer, so we may need to include more studies at a later stage to prove the conclusion. In addition, most of the studies included in this study were carried out in Asia, and such studies were carried out in other ethnic groups to observe whether the research results were different from those in Asia, which is worth exploring later.

## 5 Conclusion and prospect

In conclusion, this study confirmed the important role of circRNA in the diagnosis and prognosis of pancreatic cancer through a systematic and comprehensive search and screening of literature and analysis of data, laying a solid foundation for further basic research. At the same time, we should also realize that researchers’ research on circRNA is still at the basic stage and that there is still a long way to go to realize its clinical value, which is a huge challenge for scientific research.

## Data availability statement

The original contributions presented in the study are included in the article/**Supplementary Material**. Further inquiries can be directed to the corresponding authors.

## Author contributions

RZ and ZH wrote the draft of the manuscript. ZH, HZ, and YX were responsible for extracting data. XBC, XGC, and YX contributed to the conception to the work and the structure of the manuscript. All authors contributed to the article and approved the submitted version.
