# Supplementary material for: Diagnostic and prognostic role of circRNAs in pancreatic cancer: a meta-analysis
Source: Front Oncol. 2023 Jun 8;13:1174577. doi: 10.3389/fonc.2023.1174577 (PMC10285410; doi:10.3389/fonc.2023.1174577)
Supplement: Supplementary file 1 [file Table_1.docx]

Supplement table 1. Correlation between age and circRNA

| first author | publish year | a | b | c | d |
| --- | --- | --- | --- | --- | --- |
| Wong, C. H. | 2022 | 43 | 10 | 10 | 6 |
| Meng, L. | 2022 | 38 | 10 | 37 | 12 |
| Hong, L.01 | 2022 | 14 | 17 | 21 | 10 |
| Hong, L.02 | 2022 | 20 | 11 | 15 | 16 |
| Fu, X. | 2022 | 17 | 12 | 14 | 14 |
| Zhang, J. | 2021 | 13 | 10 | 10 | 12 |
| Xu, K. | 2021 | 24 | 19 | 11 | 8 |
| Shen, X. | 2021 | 1 | 12 | 4 | 9 |
| Shen, P. | 2021 | 34 | 18 | 37 | 15 |
| Rong, Z. | 2021 | 74 | 64 | 41 | 30 |
| Li, S. | 2021 | 16 | 3 | 7 | 4 |
| Guan, H. | 2021 | 8 | 8 | 6 | 8 |
| Zhang, X. | 2020 | 9 | 25 | 12 | 21 |
| Hou, Y. S. | 2020 | 14 | 9 | 20 | 13 |
| Guo, W. | 2020 | 14 | 13 | 10 | 17 |
| Xu, Y. | 2019 | 18 | 14 | 14 | 16 |
| Hao, L. | 2019 | 17 | 13 | 14 | 16 |
| Chen, Y. | 2019 | 21 | 24 | 20 | 25 |
| Li, Z. | 2018 | 22 | 24 | 21 | 26 |
| Li, J. | 2018 | 22 | 20 | 17 | 26 |
| Jiang, Y. | 2018 | 14 | 11 | 20 | 13 |
| An, Y. | 2018 | 39 | 44 | 18 | 15 |
| Yang, F. | 2017 | 10 | 12 | 5 | 4 |

Supplement table 2. Correlation between gender and circRNA

| first author | publish year | a | b | c | d |
| --- | --- | --- | --- | --- | --- |
| Zheng, S. | 2022 | 41 | 39 | 46 | 35 |
| Wong, C. H. | 2022 | 36 | 17 | 4 | 12 |
| Meng, L. | 2022 | 31 | 17 | 32 | 17 |
| Hong, L.01 | 2022 | 15 | 16 | 19 | 12 |
| Hong, L.02 | 2022 | 18 | 13 | 16 | 15 |
| He, Z. | 2022 | 28 | 18 | 24 | 22 |
| Fu, X. | 2022 | 11 | 18 | 10 | 18 |
| Zhang, J. | 2021 | 12 | 11 | 13 | 9 |
| Xu, K. | 2021 | 24 | 19 | 10 | 9 |
| Shen, X. | 2021 | 4 | 9 | 6 | 7 |
| Shen, P. | 2021 | 36 | 16 | 34 | 18 |
| Rong, Z. | 2021 | 75 | 63 | 30 | 41 |
| Liu, X. | 2021 | 27 | 13 | 30 | 10 |
| Li, S. | 2021 | 11 | 8 | 8 | 3 |
| Hou, J. P. | 2021 | 9 | 6 | 7 | 8 |
| Guan, H. | 2021 | 9 | 7 | 8 | 6 |
| Zhang, X. | 2020 | 18 | 16 | 20 | 13 |
| Hou, Y. S. | 2020 | 9 | 14 | 11 | 22 |
| Guo, W. | 2020 | 13 | 14 | 12 | 15 |
| Xu, Y. | 2019 | 14 | 18 | 15 | 15 |
| Hao, L. | 2019 | 12 | 18 | 15 | 15 |
| Chen, Y. | 2019 | 34 | 11 | 36 | 9 |
| Li, Z. | 2018 | 34 | 12 | 38 | 9 |
| Li, J. | 2018 | 31 | 11 | 35 | 8 |
| Jiang, Y. | 2018 | 14 | 11 | 17 | 16 |
| An, Y. | 2018 | 41 | 32 | 17 | 16 |
| Yang, F. | 2017 | 14 | 8 | 5 | 4 |

Supplement table 3. Correlation between tumor size and circRNA

| first author | publish year | a | b | c | d |
| --- | --- | --- | --- | --- | --- |
| Wong, C. H. | 2022 | 32 | 21 | 6 | 10 |
| Hong, L.01 | 2022 | 22 | 9 | 19 | 12 |
| Hong, L.02 | 2022 | 16 | 15 | 25 | 6 |
| Zhang, J. | 2021 | 10 | 13 | 13 | 9 |
| Xu, K. | 2021 | 11 | 32 | 13 | 6 |
| Shen, X. | 2021 | 8 | 5 | 7 | 6 |
| Shen, P. | 2021 | 37 | 15 | 44 | 8 |
| Hou, J. P. | 2021 | 12 | 3 | 8 | 7 |
| Hou, Y. S. | 2020 | 10 | 13 | 21 | 12 |
| Guo, W. | 2020 | 12 | 15 | 9 | 18 |
| Yang, F. | 2017 | 15 | 7 | 6 | 3 |

Supplement table 4. Correlation between lymph node metastasis and circRNA

| first author | publish year | a | b | c | d |
| --- | --- | --- | --- | --- | --- |
| Zheng, S. | 2022 | 9 | 71 | 12 | 69 |
| Wu, H.01 | 2022 | 7 | 17 | 11 | 16 |
| Wu, H.02 | 2022 | 6 | 18 | 12 | 15 |
| Wu, H.03 | 2022 | 8 | 16 | 10 | 17 |
| Wu, H.04 | 2022 | 8 | 22 | 10 | 20 |
| Wu, H.05 | 2022 | 5 | 25 | 13 | 17 |
| Wu, H.06 | 2022 | 6 | 24 | 12 | 18 |
| Wu, H.07 | 2022 | 10 | 20 | 8 | 21 |
| Wu, H.08 | 2022 | 8 | 22 | 10 | 20 |
| Meng, L. | 2022 | 16 | 32 | 28 | 21 |
| Hong, L.02 | 2022 | 21 | 10 | 24 | 6 |
| He, Z. | 2022 | 11 | 35 | 22 | 24 |
| Fu, X. | 2022 | 3 | 26 | 11 | 17 |
| Zhang, J. | 2021 | 8 | 15 | 16 | 6 |
| Xu, K. | 2021 | 28 | 15 | 18 | 1 |
| Shen, X. | 2021 | 4 | 9 | 10 | 3 |
| Shen, P. | 2021 | 21 | 31 | 26 | 26 |
| Liu, X. | 2021 | 12 | 23 | 29 | 11 |
| Li, S. | 2021 | 9 | 10 | 8 | 3 |
| Hou, J. P. | 2021 | 2 | 13 | 8 | 7 |
| Guan, H. | 2021 | 4 | 12 | 7 | 7 |
| Hou, Y. S. | 2020 | 10 | 13 | 23 | 10 |
| Guo, W. | 2020 | 9 | 18 | 17 | 10 |
| Hao, L. | 2019 | 4 | 26 | 13 | 17 |
| Chen, Y. | 2019 | 24 | 21 | 35 | 10 |
| Li, Z. | 2018 | 24 | 22 | 36 | 11 |
| Li, J. | 2018 | 23 | 19 | 32 | 11 |
| An, Y. | 2018 | 26 | 47 | 20 | 13 |

Supplement table 5. Correlation between grade of histology and circRNA

| first author | publish year | a | b | c | d |
| --- | --- | --- | --- | --- | --- |
| Zheng, S. | 2022 | 76 | 4 | 74 | 7 |
| Wu, H.01 | 2022 | 27 | 3 | 25 | 5 |
| Wu, H.02 | 2022 | 27 | 3 | 25 | 5 |
| Wu, H.03 | 2022 | 29 | 1 | 23 | 7 |
| Wu, H.04 | 2022 | 28 | 2 | 24 | 6 |
| Wu, H.05 | 2022 | 28 | 2 | 24 | 6 |
| Wu, H.06 | 2022 | 17 | 3 | 25 | 5 |
| Wu, H.07 | 2022 | 27 | 3 | 25 | 5 |
| Wong, C. H. | 2022 | 45 | 5 | 10 | 4 |
| Meng, L. | 2022 | 36 | 12 | 24 | 25 |
| Hong, L.01 | 2022 | 27 | 4 | 21 | 10 |
| Hong, L.02 | 2022 | 26 | 5 | 22 | 9 |
| Fu, X. | 2022 | 26 | 3 | 14 | 14 |
| Zhang, J. | 2021 | 14 | 9 | 6 | 16 |
| Shen, X. | 2021 | 4 | 9 | 3 | 10 |
| Shen, P. | 2021 | 18 | 34 | 21 | 31 |
| Rong, Z. | 2021 | 48 | 90 | 19 | 52 |
| Liu, X. | 2021 | 38 | 2 | 29 | 11 |
| Hou, J. P. | 2021 | 8 | 7 | 10 | 5 |
| Guan, H. | 2021 | 12 | 4 | 12 | 2 |
| Xu, Y. | 2019 | 11 | 19 | 17 | 15 |
| Hao, L. | 2019 | 14 | 16 | 12 | 18 |
| Chen, Y. | 2019 | 42 | 3 | 37 | 8 |
| Li, Z. | 2018 | 44 | 2 | 39 | 8 |
| Li, J. | 2018 | 40 | 2 | 37 | 6 |
| An, Y. | 2018 | 58 | 10 | 29 | 4 |

Supplement table 6. Correlation between TNM stage and circRNA

| first author | publish year | a | b | c | d |
| --- | --- | --- | --- | --- | --- |
| Zheng, S. | 2022 | 59 | 21 | 75 | 6 |
| Wu, H.01 | 2022 | 19 | 11 | 25 | 5 |
| Wu, H.02 | 2022 | 20 | 10 | 24 | 6 |
| Wu, H.03 | 2022 | 20 | 10 | 24 | 6 |
| Wu, H.04 | 2022 | 21 | 9 | 23 | 7 |
| Wu, H.05 | 2022 | 17 | 13 | 27 | 3 |
| Wu, H.06 | 2022 | 18 | 12 | 26 | 4 |
| Wu, H.07 | 2022 | 20 | 10 | 24 | 6 |
| Wu, H.08 | 2022 | 21 | 9 | 23 | 7 |
| Wong, C. H. | 2022 | 42 | 10 | 13 | 3 |
| Meng, L. | 2022 | 27 | 21 | 22 | 27 |
| Hong, L.01 | 2022 | 14 | 17 | 13 | 18 |
| Hong, L.02 | 2022 | 11 | 20 | 16 | 15 |
| He, Z. | 2022 | 32 | 14 | 28 | 18 |
| Fu, X. | 2022 | 22 | 9 | 26 | 2 |
| Xu, K. | 2021 | 15 | 28 | 12 | 7 |
| Shen, P. | 2021 | 14 | 38 | 21 | 31 |
| Guan, H. | 2021 | 4 | 12 | 8 | 6 |
| Guo, W. | 2020 | 11 | 16 | 21 | 6 |
| Chen, Y. | 2019 | 19 | 26 | 32 | 13 |
| Li, Z. | 2018 | 19 | 27 | 33 | 14 |
| Li, J. | 2018 | 18 | 24 | 29 | 14 |
| Yang, F. | 2017 | 19 | 3 | 9 | 0 |

Supplement table 7. Correlation between distant metastasis and circRNA

| first author | publish year | a | b | c | d |
| --- | --- | --- | --- | --- | --- |
| He, Z. | 2022 | 41 | 5 | 44 | 2 |
| Shen, P. | 2021 | 50 | 2 | 52 | 0 |
| Liu, X. | 2021 | 21 | 19 | 33 | 7 |
| Hou, Y. S. | 2020 | 9 | 14 | 23 | 10 |
